# Supplementary material for: Risk of Preeclampsia and Adverse Pregnancy Outcomes after Heterologous Egg Donation: Hypothesizing a Role for Kidney Function and Comorbidity
Source: J Clin Med. 2019 Oct 28;8(11):1806. doi: 10.3390/jcm8111806 (PMC6912476; doi:10.3390/jcm8111806)
Supplement: Supplementary file 1 [file jcm-08-01806-s001.pdf]

Table S1. Multivariate analysis of the different outcomes: all cases.

	**PREECLAMPSIA**	**Sig.**	**Exp(B)**	**CI 95% EXP(B)**	
				**Lower**	**Higher**
1st step	Age (dichotomised at the median)	0.326	1.456	0.688	3.082
	BMI (dichotomised at 30 kg·m^{-2})	0.306	1.781	0.589	5.381
	Parity (1st vs. other)	0.165	0.236	0.031	1.807
	Comorbidity (renal and other)	0.056	2.355	0.979	5.667
2nd step	BMI (dichotomised at 30 kg·m^{-2})	0.277	1.845	0.611	5.569
	Parity (1st vs. other)	0.171	0.242	0.032	1.845
	Comorbidity (renal and other)	0.061	2.310	0.961	5.550
3rd step	Parity (1st vs. other)	0.152	0.227	0.030	1.725
	**Comorbidity (renal and other)**	**0.035**	**2.513**	**1.066**	**5.923**
	**Delivery < 37 weeks**	**Sig.**	**Exp(B)**	**CI 95% EXP(B)**	
				**Lower**	**Higher**
1st step	Age (dichotomised at the median)	0.910	0.963	0.504	1.840
	BMI (dichotomised at 30 kg·m^{-2})	0.694	1.244	0.418	3.700
	Parity (1st vs. other)	0.914	0.946	0.342	2.615
	Comorbidity (renal and other)	0.023	2.477	1.136	5.398
2nd step	Age (dichotomised at the median)	0.907	0.962	0.504	1.837
	BMI (dichotomised at 30 kg·m^{-2})	0.685	1.252	0.423	3.703
	Comorbidity (renal and other)	0.021	2.487	1.145	5.402
3rd step	BMI (dichotomised at 30 kg·m^{-2})	0.689	1.247	0.423	3.682
	Comorbidity (renal and other)	0.021	2.490	1.147	5.407
**4th step**	**Comorbidity (renal and other)**	**0.015**	**2.565**	**1.198**	**5.488**
	**Small for gestational age**	**Sig.**	**Exp(B)**	**CI 95% EXP(B)**	
				**Lower**	**Higher**
1st step	Age (dichotomised at the median)	0.757	0.876	0.380	2.020
	BMI (dichotomised at 30 kg·m^{-2})	0.291	0.325	0.040	2.617
	Parity (1st vs. other)	0.484	0.586	0.131	2.622
	Comorbidity (renal and other)	0.048	2.634	1.011	6.866
2nd step	BMI (dichotomised at 30 kg·m^{-2})	0.287	0.322	0.040	2.588
	Parity (1st vs. other)	0.478	0.581	0.130	2.602
	Comorbidity (renal and other)	0.047	2.637	1.012	6.868
3rd step	BMI (dichotomised at 30 kg·m^{-2})	0.306	0.337	0.042	2.704
	Comorbidity (renal and other)	0.039	2.735	1.054	7.098
**4th step**	**Comorbidity (renal and other)**	**0.060**	**2.453**	**0.961**	**6.257**

First and last step shown in tables (complete analysis). Significant results in bold.

Table S2. Multivariate analysis of the different outcomes: cases with creatinine assessment (complete analysis).

	**PREECLAMPSIA**	**Sig.**	**Exp(B)**	**CI 95% EXP(B)**	
				**Lower**	**Higher**
1st step	Age (dichotomised at the median)	0.472	1.342	0.603	2.986
	BMI (dichotomised at 30 kg·m^{-2})	0.339	1.478	0.664	3.291
	Parity (1st vs. other)	0.312	0.340	0.042	2.756
	Comorbidity (renal and other)	0.880	1.063	0.480	2.357
	sCreat. (dichotomised at the median)	<0.001	15.809	4.631	53.968
2nd step	Age (dichotomised at the median)	0.478	1.335	0.602	2.961
	BMI (dichotomised at 30 kg·m^{-2})	0.340	1.476	0.663	3.284
	Parity (1st vs. other)	0.309	0.338	0.042	2.734
	sCreat. (dichotomised at the median)	<0.001	15.975	4.714	54.136
3rd step	BMI (dichotomised at 30 kg·m^{-2})	0.387	1.417	0.643	3.122
	Parity (1st vs. other)	0.313	0.341	0.042	2.758
	sCreat. (dichotomised at the median)	<0.001	16.191	4.783	54.808
4th step	Parity (1st vs. other)	0.315	0.343	0.042	2.764
	sCreat. (dichotomised at the median)	<0.001	16.142	4.773	54.596
**5th step**	**sCreat. (dichotomised at the median)**	**<0.001**	**17.277**	**5.125**	**58.238**
	**Delivery < 37 weeks**	**Sig.**	**Exp(B)**	**CI 95% EXP(B)**	
				**Lower**	**Higher**
1st step	Age (dichotomised at the median)	0.864	1.075	0.467	2.474
	BMI (dichotomised at 30 kg·m^{-2})	0.616	0.689	0.161	2.955
	Parity (1st vs. other)	0.972	1.031	0.186	5.711
	Comorbidity (renal and other)	0.346	1.596	0.603	4.224
	sCreat. (dichotomised at the median)	0.062	2.315	0.958	5.593
2nd step	Age (dichotomised at the median)	0.864	1.075	0.467	2.474
	BMI (dichotomised at 30 kg·m^{-2})	0.614	0.688	0.161	2.944
	Comorbidity (renal and other)	0.342	1.592	0.610	4.154
	sCreat. (dichotomised at the median)	0.062	2.317	0.960	5.590
3rd step	BMI (dichotomised at 30 kg·m^{-2})	0.605	0.682	0.160	2.912
	Comorbidity (renal and other)	0.348	1.583	0.607	4.125
	sCreat. (dichotomised at the median)	0.062	2.315	0.959	5.585
4th step	Comorbidity (renal and other)	0.396	1.502	0.587	3.845
	sCreat. (dichotomised at the median)	0.067	2.278	0.945	5.491
**5th step**	**sCreat. (dichotomised at the median)**	**0.029**	**2.545**	**1.1**	**5.892**
	**Small for gestational age**	**Sig.**	**Exp(B)**	**CI 95% EXP(B)**	
				**Lower**	**Higher**
1st step	Age (dichotomised at the median)	0.550	1.402	0.463	4.243
	BMI (dichotomised at 30 kg·m^{-2})	0.585	0.543	0.061	4.865
	Parity (1st vs. other)	0.779	1.377	0.148	12.830
	Comorbidity (renal and other)	0.082	3.038	0.867	10.644
	sCreat. (dichotomised at the median)	0.535	0.687	0.210	2.251
2nd step	Age (dichotomised at the median)	0.549	1.403	0.464	4.245
	BMI (dichotomised at 30 kg·m^{-2})	0.576	0.536	0.060	4.776
	Comorbidity (renal and other)	0.085	2.938	0.861	10.025
	sCreat. (dichotomised at the median)	0.545	0.693	0.212	2.268
3rd step	Age (dichotomised at the median)	0.503	1.457	0.484	4.381
	Comorbidity (renal and other)	0.098	2.817	0.826	9.612
	sCreat. (dichotomised at the median)	0.498	0.664	0.203	2.172
4th step	Comorbidity (renal and other)	0.106	2.750	0.805	9.393
	sCreat. (dichotomised at the median)	0.483	0.653	0.199	2.145
5th step	Comorbidity (renal and other)	0.141	2.333	0.755	7.210

Significant results in bold.
